# Supplementary material for: Fear of the unknown: Experience of frontline healthcare workers with coping strategies used to face the COVID 19 pandemic
Source: PLOS Glob Public Health. 2024 Aug 15;4(8):e0003373. doi: 10.1371/journal.pgph.0003373 (PMC11326562; doi:10.1371/journal.pgph.0003373)
Supplement: Attachment — Submitted filename: Response to Reviewers.docx [file pgph.0003373.s001.docx]

Laura Becerra Sarmiento

Pontificia Universidad Javeriana

Bogotá, Colombia

Dr. Suma Krishnasastry

Academic Editor

PLOS Global Public Health

May, 2024

Subject: Revision and resubmission of manuscript “*Fear of the unknown: Experience of frontline healthcare workers with coping strategies used to face the* *COVID 19 pandemic”*

Dear Dr. Krishnasastry,

Thank you for the opportunity to revise our paper on *Fear of the unknown: Experience of frontline healthcare workers with coping strategies used to face the* *COVID 19 pandemic”*. The suggestions offered by the reviewers have been very helpful, and we appreciate the comments on the paper.

I have included the reviewer comments immediately after this letter and responded to them individually, indicating exactly how we addressed each concern and describing the changes we have made. The revisions have been approved by all authors and I have again been chosen as the corresponding author. Additionally, as required, a marked-up copy of the manuscript highlighting the changes made to the original version has been submitted labeled as “Revised Manuscript with Track Changes”.

We hope the revised manuscript will better suits, *PLOS Global Public Health*, but are happy to consider further revisions, and we thank you for your continued interest in our research.

Sincerely,

Laura Becerra Sarmiento

Pontificia Universidad Javeriana

Bogotá, Colombia

**Journal Requirements**

**Comment:** Please review your reference list to ensure that it is complete and correct. If you have cited papers that have been retracted, please include the rationale for doing so in the manuscript text or remove these references and replace them with relevant current references. Any changes to the reference list should be mentioned in the rebuttal letter that accompanies your revised manuscript. If you need to cite a retracted article, indicate the article’s retracted status in the References list and also include a citation and full reference for the retraction notice.

**Response:** Thank you for your comment. We have revised the cited papers and did not find any which were retracted.

**Change:** Accordingly, we have maintained the same bibliography and reference list. Moreover, citations and reference list have formatted to Vancouver style.

**Comment:** We have noticed that you have uploaded Supporting Information files, but you have not included a list of legends. Please add a full list of legends for your Supporting Information files after the references list.

**Response**: Thank you for your comment. We will add the legend for clarity after the reference list.

**Change:** We added the legend for the Supporting Information files after the reference list.

**Reviewer Comments, Author Responses and Manuscript Changes**

**Reviewer 2**

**Comment to authors:**  I read this article with interest.

**Comment:** However, the sampling and collection method suffers from a few shortcomings that could be a source of bias (choice of site, criteria for including participants). The choice of study site (health centre, hospital) and the criteria for recruiting participants should be included and detailed.

**Response**: Thank you for your comment. Given that our investigation uses a qualitative methodology, we believe that convenience sampling is appropriate as noted by Moser and Korstjensc [1]. Moreover, according to our phenomenological approach, we deliberately selected participants who shared a common experience but possessed diverse characteristics such as different medical specialties or roles, marital status, age, or gender. This selection allows for a more comprehensive exploration of the impact of treating COVID-19 patients and being infected with the virus on the frontline healthcare workers’ mental health and coping strategies.

**Reviewer 3**

**Comment to authors:**

Thank you for an insightful article. A few observations were made.

**Comment**:  There was no introduction in the abstract. It began with the aims of the study.

**Response:** Thank you for your comment. We agree with you. An introduction in the abstract has been included to give readers essential context.

**Changes:** We added the introduction in the abstract and it is with tracked changes.

**Comment: T**he are some grammatical errors which will need to be worked on. The following are some of the phrases I think need to be worked on:

"... the experience of HCWs during the attention of COVID-19 patients...."
"... conducted the study during August 2021....."
"..... at the beginning they imposed a challenge....."
"Such rejection acts were primary due to the fear..."
"More sadness and impotence were also a common feeling...."
"It is clear that to attend patients is very different...."
".... return to their jobsassoon a quarantine. "
" ...being more sensible to everything. "

**Response:** Thank you for your comment. We agree with you, there were grammatical errors which have been corrected throughout the manuscript.

**Changes:** Grammatical corrections have been made, and are with tracked changes.

**References**

1.	Moser A, Korstjens I. Series: Practical guidance to qualitative research. Part 3: Sampling, data collection and analysis. Vol. 24, European Journal of General Practice. Taylor and Francis Ltd; 2018. p. 9–18.
